# The Role of Immune and Inflammatory Mechanisms in ALS

**DOI:** 10.2174/156652411795243450

**Published:** 2011-04

**Authors:** P.A McCombe, R.D Henderson

**Affiliations:** The University of Queensland, UQ Centre for Clinical Research, Australia

**Keywords:** Amyotrophic lateral sclerosis, biomarkers, immunity, inflammation, lymphocytes, protective immunity, T cells.

## Abstract

Amyotrophic lateral sclerosis (ALS) is a severe progressive neurodegenerative disease. The cause is unknown, but genetic abnormalities have been identified in subjects with familial ALS and also in subjects with sporadic ALS. Environmental factors such as occupational exposure have been shown to be risk factors for the development of ALS. Patients differ in their clinical features and differ in the clinical course of disease. Immune abnormalities have been found in the central nervous system by pathological studies and also in the blood and CSF of subjects with ALS. Inflammation and immune abnormalities are also found in animals with a model of ALS due to mutations in the SOD1 gene. Previously it has been considered that immune abnormalities might contribute to the pathogenesis of disease. However more recently it has become apparent that an immune response can occur as a response to damage to the nervous system and this can be protective.

## INTRODUCTION

Neurodegenerative diseases such as amyotrophic lateral sclerosis (ALS) lack the prominent infiltrates of blood-derived mononuclear cells that characterize primary autoimmune diseases. However, there is abundant evidence many substances involved in the promotion of inflammatory processes are present in the CNS of patients with such neurodegenerative diseases and there are also modest numbers of inflammatory cells present in the tissue in ALS. There have been previous reviews of the role of inflammation in ALS and the possibility of treating ALS by immune modulation [1-3]. We now review the evidence of immune abnormalities in ALS and whether this is helpful or harmful. 

There have been suggestions that immune modulation could be used to modify the course of ALS. However, before such therapy is attempted, it is important to know if the immune response is harmful or beneficial. The presence of antibody and T cells at a site of pathology can occur as part of a harmful autoimmune process although there are a number of other criteria that must be met for a disease to be considered autoimmune [4]. Immune abnormalities at the site of disease could also be a response to damage [5] through activation of the innate immune system. This occurs as a response to so-called “danger signals” that are molecules released from damaged tissue [6]. Release of mitochondria is known to be important in provoking a systemic response to tissue injury [7]. In the brain the cells that respond to damage are microglia [8]. After an initial innate immune response due to microglia there can be further adaptive immune response to injury.

Once it has been provoked, an immune response could modulate the rate of progression of disease. One possibility is that an immune response could make disease more severe. However, an immune response can also be protective [9] and indeed strategies that enhance protective immunity are possible options for therapy of neurological diseases [10]. We will review the clinical features and pathogenesis if ALS, then provide evidence of immune abnormalities in ALS and the evidence for a role of these in pathogenesis and disease progression, including studies in experimental animal models of ALS.

## BACKGROUND TO ALS

### Clinical Features

ALS is a progressive disorder causing weakness of the limbs, and leading to death, usually within 3 –5 years. The incidence of ALS is around 2 per 100,000 [11-15]. ALS is slightly more common in men than in women [16]. For the diagnosis of ALS, there are strict clinical definitions [17] that involve the finding of a combination of upper and lower motor neurone signs. At first presentation, some patients do not fulfil these strict criteria but as time goes by they develop additional signs that confirm the diagnosis [18]. Although predominantly a motor disorder, ALS is commonly associated with dementia [19]. Although not clinically apparent, testing had found that there can also be subtle sensory abnormalities [20] and autonomic dysfunction [21]. 

### Sub-Types of Disease

Patients with ALS vary in their clinical features such as the site of onset, and whether the subject has features of both upper and lower motor neurone weakness, or has solely upper or lower motor neurone signs. Patients with ALS may be placed into further subgroups (phenotypes) by combining information about both the site of onset (bulbar; upper or lower limb) and the type of weakness (predominantly affecting the upper or lower motor neurons). This type of analysis identifies groups such as the flail-arm presentation, which involves lower motor neuron weakness of the upper limbs [22, 23]. Cluster analysis has also found distinct subgroups of patients [23]. Furthermore the gender of the subject influences the site of onset of ALS, with women being more likely to have bulbar onset disease [16]. It is possible that the different sub-types of ALS have different pathogenesis. In one of the genome-wide association studies in ALS, the investigators found that the association with different SNPs was different according to gender [24]. It is important to understand the causes of heterogeneity in ALS, because sub-types of disease with different pathogenesis could confound clinical trials [25]. It is possible that the immune response could be important in the disease process, and this could vary among individuals, leading to further heterogeneity. 

### Prognostic Factors

In addition to different clinical features, patients also vary in the rate of progression of weakness and it may be that once the disease develops, there are factors that influence the rate of progression. The known prognostic factors in ALS are age and bulbar site of onset [26]. It is possible that gender also plays a role in prognosis [26] with women having a shorter survival on average, but this is controversial, and gender does not appear to be an independent risk factor in multivariate analysis [16]. It is important to look for modifiable factors that affect prognosis, because this could lead to possible therapies. This review will focus on the role of the immune system and whether immune responses alter the rate of progression of disease. Males and females have different immune responsiveness [27], so this could be another variable that could lead to differences between men and women in the clinical course of ALS.

### Measuring Disease Progression in ALS

While prolonging survival of patients with ALS is the goal of therapy, survival time is not a good measure of the underlying rate of progression of disease, because survival is affected by other factors such as the use of mechanical ventilation [28], and also the site of onset of disease, so that subjects with early involvement of respiratory muscles have a shorter survival [29]. The underlying rate of death of upper and lower motor neurones is more difficult to measure. For this we need biomarkers which are “objective measurements that act as an indicator of normal biological processes, pathogenic processes or pharmacological response to therapeutic intervention” [30]. Any studies of the rate of disease progression in ALS need to measure the rate of cell death or surrogate markers of this. One measure of disease progression in ALS is motor unit number estimation, using neurophysiological techniques to determine the number of motor units in a muscle [30, 31]. We have developed a method of motor unit number estimation that uses measurement of the compound muscles action potential in response to increasing levels of electrical stimulation. This data is analyzed using Bayesian statistics to provide an estimate of the number of motor units in the muscle [32, 33]. This can be used repeatedly to measure the number of motor units in a muscle so that the rate of loss of motor units can be calculated [34]. Other possible biomarkers include serum levels of neurofilaments which are a measure of axonal degeneration and which are elevated in ALS [35]. 

## BACKGROUND TO PATHOGENESIS OF ALS

The pathology of ALS includes loss of both upper and lower motor neurones, but the fundamental processes that lead to the death of neurones are also not fully understood [36] . Theories of the pathogenesis include the effects of abnormal proteins, such as TDP-43 [37, 38] , altered mitochondrial dysfunction [39], and glutamate toxicity [36]. Numerous studies have demonstrated biochemical abnormalities in autopsy tissue including AMPA receptor medicated toxicity [40], increased cytosolic phospholipase A(2) [41] and activation of apoptosis inducing factor [42]. While ALS is primarily a disease of motor neurones, there is also damage that is dependent on factors external to the motor neurone. Astrocytes have been implicated in causing such damage [43]. This is known as non-cell autonomous damage [44, 45] and occurs in other neurodegenerative diseases as well as in ALS. 

It is likely that genetic and environmental factors play a role in pathogenesis of ALS. Some cases are familial ALS (fALS) with a number of causative genes being identified. The first gene to be identified was Cu/Zn superoxide dismutase 1 (SOD1), which has numerous different mutations [46]. The exact means of toxicity of mutant SOD1 is not fully understood, but mutant SOD1 expression in astrocytes and microglia contributes to disease progression in ALS [45]. Other genes implicated in fALS are fused in sarcoma protein (FUS) [47] and tar-DNA binding protein of molecular weight 43kDa (TDP 43) [48]. 

In sporadic ALS, genetic factors are also important. Having even a single relative with ALS increases risk of disease for an individual [49]. The genes implicated in sporadic ALS (sALS) include TDP-43 [48], FUS [50] and the SMN gene [51]. There have been several genome wide association studies in ALS. These have found somewhat differing results [24, 52, 53] but have found associations with genes for neurotransmitter release, genes associated with familial spastic paraparesis and genes associated with frontotemporal dementia. Environmental factors linked to ALS include cigarette smoking [54], occupational exposures particularly to toxins and metals, exercise [55, 56] and education [57-60]. 

## EVIDENCE OF THE PRESENCE OF IMMUNE AND INFLAMMATORY ABNORMALITIES IN ALS

### Findings from Pathology and Imaging

#### Studies in Humans

There is considerable evidence of inflammation in ALS. Studies of human post-mortem pathology have shown immune abnormalities in ALS. However, these studies are necessarily studies done at the end stage of disease, and do not reveal the early changes. In studies of ALS pathology there is morphological evidence of microglial activation [61-63]. Microglial activation occurs after tissue injury and involves change in shape and expression of cell surface receptors [8, 64] and is part of an innate immune response. Microglial activation in ALS has been further demonstrated by the finding of the signal transducer and activator of transcription-3 (STAT3) in microglia in autopsy studies [65]. Gene expression studies have found upregulation of the TLR4 signalling genes in subjects with ALS [66] and the authors suggest that this indicates chronic monocyte/macrophage activations. 

Nuclear medicine technology is able to demonstrate neuroinflammation [67] and microglial activation can be demonstrated by binding of the PK 11195 ligand to the peripheral benzodiazepine receptor [68, 69]. Using this ligand, microglial activation has been demonstrated in ALS with PET imaging [70]. In ALS it is not clear is whether this change occurs early in disease or is a reaction to disease. For example, microglia activation can occur after distant pathology such as after a dying-back axonopathy [71]. 

There are also infiltrating immune cells in the CNS in human ALS [72] . These include macrophages and mast cells [73] and also T cells in the areas of motor neuron destruction [63, 74, 75]. There is evidence of immunoglobulin deposition in the CNS in ALS [76] and also of complement deposition [63, 77]. 

#### Studies in Animal Models

The most commonly used animal models of ALS are rats or mice with mutant SOD1. In human ALS, SOD1 mutations only account for a small proportion of subjects and the mechanisms of disease may differ from other subjects in whom abnormalities of TDP-43 are found [78]. However, animals with SOD1 mutations show progressive weakness typical of ALS. In symptomatic SOD1 mutant animals, there is evidence of immune activation, although the timing of onset of inflammation is not clear. One study showed that in G93A SOD1 mutant mice, early disease is associated with astrogliosis and late disease with microglial activation [79] while another suggested that microglial activation was an early event [80]. T cells are also found in the nervous system of mice with the SOD1 mutation [81]. Inflammation in SOD1 mutant mice is associated with activation of caspase 1 and caspase 3 [82]. 

### Immune Abnormalities in Blood and CSF in ALS

There have been numerous studies investigating peripheral immune abnormalities in ALS. These include studies of antibodies, T cells, chemokines and cytokines and other markers of inflammation. The first studies were concerned with the presence of antibodies in the blood of subject with ALS. There have been many reports of antibodies to voltage gated calcium channels [83, 84]. In addition there have been studies of non-specific changes in antibodies, as a recent study has shown an increase in IgG levels in subjects with ALS compared to controls [85]. 

With respect to T cell abnormalities, in the blood of subjects with ALS, there have been reports of increased numbers of CD4+ T helper cells and increased expression of HLA class II molecules on monocytes and macrophages, suggestive of systemic immune activation [86]. Another study also found increased CD4^+^ cells, reduced regulatory T cells (Treg) but reduced expression of HLA DR by monocytes [87]. T cell clones from CSF of ALS subjects can be induced to secrete IFN gamma [75]. IL-13 producing T cells have been found in the blood of subjects with ALS and correlate with the rate of disease progression [88, 89]. The co-stimulatory pathway activated through CD40 ligand is upregulated in some human subjects with ALS [90]. 

There are increased levels of circulating chemokines and cytokines in ALS. There are higher levels of the chemokine MCP-1 in patients with a shorter diagnostic delay, which is a marker of more severe rapidly progressing disease [91]. Expression of MCP-1 receptor (CCR2) is reduced on circulating monocytes in ALS [92]. Increased levels of IL17 are found in the serum of subjects with ALS [88]. Levels of IL-6 are elevated in ALS, but only in subjects with hypoxia, so are probably a response to hypoxia rather than to the disease itself [93]. 

Other markers of inflammation are also abnormal in ALS. Levels of lipopolysaccharide are elevated in patients with ALS suggesting systemic inflammation [94]. There are also abnormalities of complement in ALS. Two dimensional gel electrophoresis was used to study serum proteins in ALS subjects and found that components of complement C3 were increased compared to controls [95]. There is also evidence of low level systemic inflammation with increased levels of C reactive protein and ESR in subjects with ALS compared to controls, with the levels correlating the levels of disability as measured by the ALS functional rating scale [96]. All these studies demonstrate the presence of an immune response in subjects with ALS. 

## DOES INFLAMMATION PARTICIPATE IN PATHOGENESIS?

### The Immune Response can be Helpful or Harmful

Having shown that there is local and systemic alteration in the immune system in ALS, it is necessary to determine if this primary or secondary, and whether it is harmful or beneficial. The immune and inflammatory changes in ALS could be primary and part of the cause of the disease. Alternatively neuroinflammation and T cell infiltration could also be secondary to the tissue damage that occurs in ALS, as it is in other nervous system injury. Once established, inflammation and immune changes could exacerbate damage [97] or be protective [98]. The protective aspects of inflammation include clearance of debris by microglia which is important in repair [99] and interaction with T cells [98]. Brain-specific T cells at the site of injury can play a role in the repair of damaged or inflamed tissues — this has been termed “protective immunity” [9, 100, 101]. This is likely to be due to the effects of cytokines and growth factors delivered by T cells to the site of injury [102-105]. Such protective immunity was appears to be a general phenomenon, that is homeostatic [106]. 

To determine whether the immune system contributes to disease it is necessary to look at the effects of passive transfer, experimental studies in animals and the effects of modifying the immune response in humans with ALS. 

### Tissue Culture and Passive Transfer of Disease

In older studies, immunoglobulins from patients with ALS were toxic to motor neurones in culture, and thought to act on calcium channels. Passive transfer to mice of ALS immunoglobulin caused some abnormalities at motor end-plates [107] and also caused degeneration of motor neurones after passive transfer to BalbC mice [108]. IgG from subjects with ALS caused apoptosis of neurones in primary spinal cord cultures [109]. This suggested that antibody could contribute to disease pathogenesis. 

### Experimental Studies in Animal Models 

Much of this work has been done in mice that have abnormalities of SOD1. Mutations in this gene are found in a small percentage of subjects with human ALS, so whether these results can be generalized to all subjects with ALS in unclear. There is conflicting evidence about whether the immune system is beneficial or harmful in this model. It must be noted that most mice used with the SOD1 mutation are of the BL6 strain. Mouse strains vary in their immune response. The immune system of BL6 mice produces a predominant Th1 response, as demonstrated by the response to Leishmania infection [110]. The macrophages of BL6 mice are involved in this process [111]. 

Mice with different genetic background to the SOD1 mutation have a different clinical course of disease, with SJL/J mice that are very susceptible to autoimmune disease having a shorter survival than mice with the BL6 background [112] and mice with ALR, NOD.Rag1KO and C3H background also showing a more severe phenotype than BL6, B10, BALB/c and DBA strains [113]. This has implications. It means that the results in SOD1 mice cannot necessarily be generalized to humans with ALS for two reasons- firstly not all subjects have SOD1 mutations and secondly, like different mouse strains, human ALS suffers will have different immune system capability. 

There is evidence that the immune system can exacerbate disease in SOD1 mutant mice. Non-specific inflammation seems to make disease worse. For example in SOD1 mice, endotoxemia can stimulate disease in mice [114]. The role of microglial activation appears to be complex, but there is evidence that activation of innate immunity through TLR4 activates microglia and leads to increased neurodegeneration [115]. In SOD1 mice there have been studies suggesting that treatment with minocycline, starting in the presymptomatic stage, with the intention of reducing micgroglial activation, is helpful in reducing progression [116]. However, a more recent study shows that when treatment starts in the late stages of disease, treatment with minocycline increases microglial activation [117]. In SOD1 mutant mice, treatment with bee venom led to reduction in microglial activation and with some reduction in severity of disease [118]. However, it must be noted that if microglial activation is secondary to degeneration, then any treatment that slowed degeneration would also slow microglial activation. 

Inflammation in SOD1 mice is mediated through inflammasomes, which are activated by NOD-like receptors in response to danger signals [119] and which contribute to sterile inflammation. This type of inflammation is thought to characterize autoinflammatory disorders, which are a rather new class of disorders where the clinical features include recurrent inflammation [120]. This leads to activation of caspase 1, which leads to activation of IL-1β. In SOD1 transgenic mice, mutant SOD1 leads to activation of this pathway. SOD1 mice that are deficient in caspase 1 or in IL-1β or treated with IL-1 receptor antagonists have increased lifespan [121]. SOD1 mice given intraventricular injections of a caspase inhibitor also have reduced disease severity. Taken together, these studies indicate that this type of inflammation leads to more severe disease in SOD1 1. It is not known if this mechanism is active in human subjects with ALS who have normal SOD1 protein. 

Other evidence that immune processes are harmful include the finding that inhibition of C5a ameliorates disease in SOD1 mice [122]. A monoclonal antibody to CD40 ligand, to block co-stimulation, also led to reduced weight loss in SOD1 mice [90]. 

However, while the immune system can worsen disease, it is not the primary cause of disease since SOD1 mice deficient in B cells still get disease [123] and SOD1 mice without microglia have the same disease as those with normal microglia [124]. 

There is also evidence that protective immunity can lessen the disease. Protective immunity can lessen the harmful effects of damage to the nervous system [9, 125]. Protective immunity is mediated by regulatory T cells (Treg) and transfer of wild type Treg cells delayed symptom onset in G93A SOD1 mice [126]. Vaccination with SOD1 protein induces protective immunity and lessens disease [127]. SOD1 mice that are deficient in T cells have greater progression of disease and lack the upregulation of IGF-1 and downregulation of IL-6 that are seen in control mice [128] or increased levels of pro-inflammatory cytokines and NOX2 [129] . 

### Effects of Immune Therapies in Human Subjects with ALS

It is attractive to consider that modulation of the immune response will be a useful therapy in ALS. If neuroinflammation enhances disease activity, then control of neuroinflammation should be helpful [130] possibly by enhancing protective immunity [131]. So far there is little evidence in human subjects regarding the effects of enhancing protective immunity. There have been trials of immune suppression in ALS. After treatment with minocycline, to reduce microglial activation, patients did worse [132]. This might suggest that microglial activation is beneficial in ALS. Total body irradiation and stem cell therapy were of no benefit in ALS [133]. Earlier attempts at immune therapy included treatment with intravenous immunoglobulin, which was of no benefit [134], with cyclophosphamide, which also was of no benefit [135] and with azathioprine and prednisone which was of no benefit [136]. 

Recently a trial of granulocyte colony stimulating factor led to a decrease in levels of MCP-1 and IL-17 in subjects with ALS [137]. It remains to be determined whether this will lead to clinical benefit.

### Do Genes for Immunity Modulate Human ALS?

We speculate that if the immune response plays a role in ALS, then genetic differences in immune responsiveness could affect the outcome. So far, genetic studies in ALS have concentrated on genes that are risk factors for acquiring the disease, and one recent estimate from twin studies is that the hereditable component is 61% of the risk of acquiring ALS, and the environment component is 39% [138]. Less is known about genes that modify the course of disease, although it is likely that there are such genes. The variability in the clinical course among individuals in families with FALS is evidence of this. Disease modifying genes could include genes that are protective against neurodegeneration. However, if the immune response modifies the clinical course of ALS, then genes for immune function might also be important in modifying disease. In diseases of autoimmune etiology such as multiple sclerosis, genome wide association studies show strong association with the MHC region and other immune genes [139] but this is not the case in ALS, although as already mentioned such genes are likely to influence the clinical course of ALS, rather than to be risks for acquiring ALS. We suggest that this is a field worth further study.

## SUMMARY AND FUTURE DIRECTIONS

Immune activation in the CNS can be detected in ALS and indeed in other neurodegenerative diseases such as Alzheimer’s disease [140] and Parkinson’s disease [141], where there is similar debate as to whether the immune response is helpful or harmful. Immune activation also occurs after brain injury, such as stroke, where there is also uncertainty as to whether the immune response contributes to damage or to recovery [142]. Previously it was thought that this inflammation may contribute to pathogenesis of ALS. However, it may also be a protective response. The difference in the overall effect of immune activation may be related to the timing of the response. It is possible that individual variability in immune responsiveness means that individual patients have different immune responses in ALS. What is needed now are studies that use robust measurements of disease progression to see if patients with evidence of immune activation have different prognosis from those who do not, and to explore whether disease modifying genes include genes with immune function. It is important to know whether inflammation and immune response are helpful or harmful in ALS, so that possible immunomodulatory therapies can be pursued. 
